# Suppressor of Gamma Response 1 Modulates the DNA Damage Response and Oxidative Stress Response in Leaves of Cadmium-Exposed *Arabidopsis thaliana*

**DOI:** 10.3389/fpls.2020.00366

**Published:** 2020-04-03

**Authors:** Sophie Hendrix, Verena Iven, Thomas Eekhout, Michiel Huybrechts, Ingeborg Pecqueur, Nele Horemans, Els Keunen, Lieven De Veylder, Jaco Vangronsveld, Ann Cuypers

**Affiliations:** ^1^Environmental Biology, Centre for Environmental Sciences, Hasselt University, Diepenbeek, Belgium; ^2^Department of Plant Biotechnology and Bioinformatics, Ghent University, Ghent, Belgium; ^3^Center for Plant Systems Biology, VIB, Ghent, Belgium; ^4^Biosphere Impact Studies, Belgian Nuclear Research Centre (SCKCEN), Mol, Belgium

**Keywords:** *Arabidopsis thaliana*, cadmium, DNA damage response, oxidative stress response, SIAMESE-related, suppressor of gamma response 1

## Abstract

Cadmium (Cd) exposure causes an oxidative challenge and inhibits cell cycle progression, ultimately impacting plant growth. Stress-induced effects on the cell cycle are often a consequence of activation of the DNA damage response (DDR). The main aim of this study was to investigate the role of the transcription factor SUPPRESSOR OF GAMMA RESPONSE 1 (SOG1) and three downstream cyclin-dependent kinase inhibitors of the SIAMESE-RELATED (SMR) family in the Cd-induced DDR and oxidative challenge in leaves of *Arabidopsis thaliana*. Effects of Cd on plant growth, cell cycle regulation and the expression of DDR genes were highly similar between the wildtype and *smr4/5/7* mutant. In contrast, *sog1-7* mutant leaves displayed a much lower Cd sensitivity within the experimental time-frame and significantly less pronounced upregulations of DDR-related genes, indicating the involvement of SOG1 in the Cd-induced DDR. Cadmium-induced responses related to the oxidative challenge were disturbed in the *sog1-7* mutant, as indicated by delayed Cd-induced increases of hydrogen peroxide and glutathione concentrations and lower upregulations of oxidative stress-related genes. In conclusion, our results attribute a novel role to SOG1 in regulating the oxidative stress response and connect oxidative stress to the DDR in Cd-exposed plants.

## Introduction

Cadmium (Cd) is known to indirectly increase reactive oxygen species (ROS) production in plants, thereby causing oxidative stress (Rodríguez-Serrano et al., 2006; Cuypers et al., 2011). Although ROS fulfil important roles as signaling molecules, they can damage macromolecules such as proteins, lipids and DNA in cells when present in high concentrations (Gallego et al., 2012; Cuypers et al., 2016). In order to protect themselves against oxidative stress, plants possess an extensive antioxidative defense system, consisting of both enzymatic and non-enzymatic antioxidants. An important non-enzymatic antioxidant in plants is glutathione (GSH), which functions in reducing hydrogen peroxide (H_2_O_2_) and is also involved in Cd chelation (Jozefczak et al., 2012).

As a consequence of increased ROS production, Cd exposure was reported to cause DNA damage in a variety of plant species, including *Arabidopsis thaliana* (Wang et al., 2016; Cao et al., 2018), *Triticum vulgare* (Mutlu and Mutlu, 2015), *Oryza sativa* (Zhang et al., 2015), *Allium cepa* and *Lactuca sativa* (Silveira et al., 2017). When cells perceive DNA damage, the activity of cyclin-dependent kinases (CDKs) can be affected, thereby inhibiting cell cycle progression (Hu et al., 2016). During the classical cell cycle, CDK activity reaches two threshold levels: one for DNA replication and one for mitosis. In addition to the classical cell cycle, plant cells can also undergo endoreplication (also termed “endoreduplication”), a process that is involved in plant growth and development but is also often induced during stress conditions (De Veylder et al., 2011). During this alternative cell cycle mode, CDK activity only reaches the threshold for DNA replication but not that for mitosis. As a result, nuclear DNA is replicated without subsequent cell division, resulting in endopolyploidy. The reduced mitotic CDK activity can be accomplished in three ways: (1) transcriptional downregulation of mitotic cyclins and CDKs, (2) proteasome-mediated degradation of mitotic cyclins, and (3) inhibition of CDK activity by CDK inhibitors (De Veylder et al., 2011).

Plant CDK inhibitors are classified into two families: the KIP-RELATED PROTEINS (KRPs) and SIAMESE-RELATED (SMR) proteins. Whereas SMRs appear to be plant-specific, KRPs display limited sequence identity with CDK inhibitors from other organisms. To date, 7 KRPs and 17 SMR proteins have been identified in *A. thaliana*. Some SMR proteins have been suggested to play a role in the DNA damage response (DDR) (Yi et al., 2014; Kumar and Larkin, 2017). Yi et al. (2014) demonstrated that exposure to hydroxyurea induced DNA double strand breaks (DSBs), likely through induction of ROS, and increased *SMR5* and *SMR7* transcript levels in *A. thaliana* roots. This transcriptional induction of *SMR5* and *SMR7* depends on SUPPRESSOR OF GAMMA RESPONSE 1 (SOG1), a NAC [no apical meristem (NAM), *A. thaliana* transcription activation factor (ATAF1/2) and cup-shaped cotyledon (CUC2)] transcription factor, which is phosphorylated and thereby activated by the cell cycle checkpoint kinase ATAXIA-TELANGIECTASIA MUTATED (ATM) in response to the detection of DSBs (Yoshiyama et al., 2013). This plant-specific transcription factor plays a key role in the plant DDR and its function strongly resembles that of the human p53, as it regulates various processes including cell cycle progression, DNA repair and programmed cell death (Yoshiyama, 2015; Ogita et al., 2018). Furthermore, SOG1 can also be phosphorylated by ATM AND RAD3-RELATED (ATR), activated in response to single strand breaks (SSBs) and stalled replication forks (Sjogren et al., 2015). In addition to *SMR5* and *SMR7*, *OXIDATIVE SIGNAL INDUCIBLE 1* (*OXI1*) was shown to be a direct target gene of SOG1 (Ogita et al., 2018). Interestingly, OXI1 plays a key role in the oxidative signaling pathway induced in *A. thaliana* upon Cd exposure (Smeets et al., 2013; Schellingen et al., 2015b). Through activation of MAP kinases (MPKs), OXI1 enhances the expression and activity of ethylene biosynthesis enzymes, thereby contributing to the fast increase in ethylene release observed in plants acutely exposed to Cd (Schellingen et al., 2014; Schellingen et al., 2015b).

As results of our previous study have shown that Cd exposure significantly inhibited both cell division and endoreplication and increased expression levels of several direct SOG1 target genes, including *SMR4*, *SMR5* and *SMR7* in *A. thaliana* leaves (Hendrix et al., 2018), the main hypothesis of this study is that SOG1 mediates the Cd-induced inhibition of cell cycle progression in leaves. Furthermore, we also aim to determine the involvement of SMR4, SMR5 and SMR7 in this response. To this end, Cd-induced responses are compared between leaves of wild-type (WT), *smr4/5/7* and *sog1-7* knockout *A. thaliana* plants. Cadmium-induced effects on cell cycle progression, DNA repair and programmed cell death – all known to be regulated by SOG1 – are determined and compared between leaves of all genotypes. In addition, the involvement of SOG1 in the Cd-induced oxidative stress response is analyzed.

## Materials and Methods

### Generation of the *smr4/5/7* Mutant

A construct targeting two distinct sites in the *SMR4* gene (*AT5G02220*) was designed using the pEn-Chimera and pDe-Cas9 vectors as described by Fauser et al. (2014). The resulting construct was transformed into *Arabidopsis smr5/7* mutant plants (Yi et al., 2014) using the floral dip method (Clough and Bent, 1998). Primary transformants were selected on agar plates containing kanamycin and propagated in the greenhouse, after which kanamycin-sensitive T2 plants were selected and genotyped at the *SMR4* locus using Sanger sequencing. Oligos to generate the construct and primers for sequencing are listed in Supplementary Table S1.

### Growth Conditions and Cadmium Exposure

Wild-type, *smr4/5/7* and *sog1-7* (Sjogren et al., 2015) mutant *A. thaliana* seeds (ecotype Col-0) were surface-sterilized and cultivated in a hydroponic system using a modified Hoagland solution (Smeets et al., 2008). Growth conditions were set as previously described (Keunen et al., 2011). At day 14 or day 19 after sowing, plants were either exposed to 5 μM CdSO_4_ via the roots or further grown under control conditions. Individual leaves or complete leaf rosettes were harvested after 24 h, 72 h or 8 days of exposure. Unless otherwise stated, samples were snap-frozen in liquid nitrogen and stored at −80°C until further analysis. Supplementary Figure S1 provides a visual representation of the different Cd exposure set-ups employed in this study. The mutant genotype of *smr4/5/7* and *sog1-7* plants was verified as described in Supplementary Table S2.

### Flow Cytometric Analysis of Endoreplication

To determine the extent of endoreplication, nuclear ploidy levels were determined in individual leaves using the CyStain^®^ PI Absolute P kit (Sysmex Partec, Görlitz, Germany). Before harvest, leaves were scanned using a conventional flatbed scanner to enable determination of leaf surface area in ImageJ (Schneider et al., 2012). Sample preparation and flow cytometric analysis were performed as described by Hendrix et al. (2018). Data were analyzed using FCS Express 4 software (De Novo Software, Pasadena, CA, USA). The endoreplication index indicating the average number of endocycles per cell was calculated as follows: [(0 x % 2C) + (1 x % 4C) + (2 x % 8C) + (3 x % 16C) + (4 x % 32C)] / 100 (Cookson et al., 2006; Boudolf et al., 2009).

### Determination of Element Concentrations

In order to determine leaf Cd concentrations, complete leaf rosettes were harvested and rinsed using distilled water. Subsequently, samples were oven-dried at 80°C and digested in HNO_3_ (70–71%) in a heat block. Cadmium concentrations in the leaf extracts were determined using inductively coupled plasma-optical emission spectrometry (ICP-OES 710, Agilent Technologies, Santa Clara, CA, USA).

### Gene Expression Analysis

To determine gene expression levels in complete rosettes, frozen samples were first ground using two stainless steel beads in the Retsch Mixer Mill MM 400 (Retsch, Haan, Germany). RNA extraction and cDNA synthesis were performed as previously described (Hendrix et al., 2018). Quantitative real-time PCR (qPCR) was performed using the 7500 Fast Real-Time PCR System (Applied Biosystems, Thermo Fisher Scientific, Waltham, MA, USA) and the Quantinova^TM^ SYBR^®^ Green PCR Kit (Qiagen, Hilden, Germany). Reactions contained 2 μL diluted cDNA sample, 5 μL Quantinova^TM^ SYBR^®^ Green dye, 0.05 μL ROX reference dye, 2.35 μL RNase-free H_2_O and forward and reverse primers (300 nM each, unless stated otherwise in Supplementary Table S3) in a total reaction volume of 10 μL. Amplification occurred at the following cycling conditions: 2 min at 95°C, 40 cycles of 5 s at 95°C and 25 s at 60°C. A dissociation curve was generated to confirm product specificity. Relative gene expression levels were determined via the 2^–ΔCq^ method and normalized against the expression of at least three stably expressed reference genes, selected using the GrayNorm algorithm (Remans et al., 2014). Forward and reverse primer sequences are shown in Supplementary Table S3. Supplementary Table S4 shows the qPCR parameters according to the Minimum Information for publication of qPCR Experiments (MIQE) guidelines (Bustin et al., 2009).

### Determination of Glutathione Levels and Redox State

Concentrations of total GSH and glutathione disulfide (GSSG) were determined in complete leaf rosettes using a plate reader method for measuring redox couples adapted from Queval and Noctor (2007), as described by dos Reis et al. (2018).

### Determination of Hydrogen Peroxide Levels

Relative H_2_O_2_ levels were determined in complete leaf rosettes using the Amplex^TM^ Red Hydrogen Peroxide/Peroxidase Assay Kit (Invitrogen, Thermo Fisher Scientific), as previously described by Hendrix et al. (2018).

### Determination of Lipid Peroxidation Levels

To determine the extent of lipid peroxidation, the concentration of thiobarbituric acid-reactive metabolites (TBArm) was determined in complete leaf rosettes. Samples were homogenized in 1 mL 0.1% trichloroacetic acid (TCA) using mortar and pestle. After centrifugation (20 000 *g*; 10 min; 4°C), 400 μL supernatant was added to 1 mL 0.5% TBA and samples were incubated at 95°C for 30 min, quickly cooled down and again centrifuged. The absorbance of the supernatant at 532 nm was determined and corrected for non-specific absorbance at 600 nm. All samples were analyzed *in duplo*.

### Statistical Analysis

All statistical analyses were performed in R version 3.3.1 (R Core Team, 2019). Normal distribution and homoscedasticity of the data were verified using the Shapiro-Wilk and Bartlett’s test, respectively. When these assumptions were not met, data were transformed (square root, inverse, exponent, logarithm). Gene expression data were standardly log-transformed. Depending on the number of variables, data were statistically analyzed using either a one-way or two-way ANOVA within each time point, followed by a *post hoc* Tukey-Kramer test to correct for multiple comparisons. If the (transformed) data did not meet the normality assumption, a non-parametric Kruskal-Wallis test was used, followed by the Wilcoxon rank sum test. Outliers were determined using the Extreme Studentized Deviate method (GraphPad Software, San Diego, CA, USA) at significance level 0.05.

## Results

Cadmium is well known to disturb plant growth and development and was shown to significantly inhibit cell division as well as endoreplication in *A. thaliana* leaves in a time-dependent manner (Hendrix et al., 2018). Furthermore, previous research demonstrated that the expression of *SMR4*, *SMR5* and *SMR7* was strongly induced in *A. thaliana* leaves upon Cd exposure (Hendrix et al., 2018). Via ChIP sequencing, these genes were shown to be direct target genes of the transcription factor SOG1 (Bourbousse et al., 2018; Ogita et al., 2018). Therefore, this study aimed to investigate the involvement of these plant-specific CDK inhibitors and their upstream regulator SOG1 in Cd-induced plant responses. To this end, responses were compared between leaves of WT, *smr4/5/7* triple knockouts and *sog1-7* mutant *A. thaliana* plants grown in hydroponics and exposed to 5 μM Cd in a short-term (24 and 72 h) and long-term (8 days) set-up (Supplementary Figure S1).

### Leaf Growth of the *sog1-7* Mutant Is Not Affected by Cadmium Exposure

**FIGURE 1:**
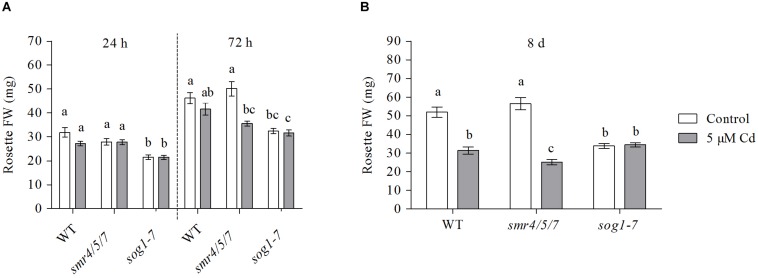
Rosette fresh weight (mg) of hydroponically grown wild-type (WT), *smr4/5/7* and *sog1-7* mutant *A. thaliana* plants exposed to 0 (white bars) or 5 μM CdSO_4_ (gray bars) for **(A)** 24 h or 72 h from day 19 after sowing or **(B)** 8 days from day 14 after sowing. Values represent the average ± S.E. of at least 9 biological replicates. Different letters indicate significant differences between conditions (2-way ANOVA per exposure duration; *P* < 0.05).

Leaf fresh weight (FW) of the *smr4/5/7* mutant did not differ from that of WT plants grown under control conditions (Figure 1). In contrast, leaf FW of *sog1-7* mutant plants was significantly lower (approximately 1.5-fold) as compared to that of WT plants. The FW of both WT and *smr4/5/7* leaves was negatively affected by exposure to 5 μM Cd, a response which was most pronounced after 8 days. However, rosette weight of *sog1-7* mutants was not affected by Cd exposure at any of the time points studied (Figure 1 and Supplementary Table S5), suggesting that this genotype is less sensitive to Cd than WT plants within the experimental time frame.

Rosette diameter and the surface area of leaves 1 (the oldest rosette leaf) and 3 were compared between plants of all genotypes grown under control conditions or exposed to 5 μM Cd for 8 days. Whereas leaf 1 was already present at the start of Cd exposure, leaf 3 had only just emerged when exposure was initiated. Results show a pattern highly similar to that observed for leaf FW. Indeed, rosette diameter and the surface area of the leaves analyzed of *sog1-7* mutants were significantly smaller as compared to those of WT plants grown under control conditions, whereas they were highly similar between *smr4/5/7* mutants and WT plants (Table 1). Furthermore, Cd exposure negatively affected rosette diameter and leaf surface area of WT and *smr4/5/7* plants, whereas this response was generally absent in *sog1-7* mutants (Table 1), again pointing toward a lower Cd sensitivity of this genotype.

**TABLE 1:**
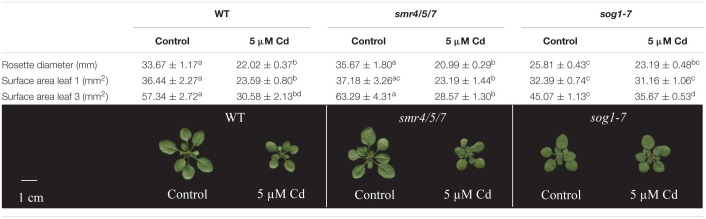
Rosette diameter (mm), surface area (mm^2^) of leaves 1 and 3 and representative pictures of hydroponically grown wild-type (WT), *smr4/5/7* and *sog1-7* mutant *A. thaliana* plants exposed to 0 or 5 μM CdSO_4_ for 8 days from day 14 after sowing.

*Values represent the average ± S.E. of 8 biological replicates. Different letters indicate significant differences between conditions (2-way ANOVA; P < 0.05).*

### Cadmium-Induced Effects on Endoreplication Are Less Pronounced in Leaves of the *sog1-7* Mutant

**FIGURE 2:**
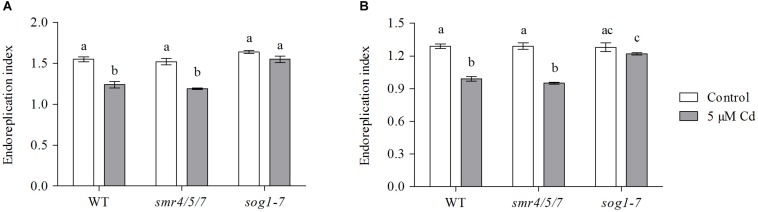
Endoreplication index of **(A)** leaf 1 and **(B)** leaf 3 of hydroponically grown wild-type (WT), *smr4/5/7* and *sog1-7* mutant *A. thaliana* plants exposed to 0 (white bars) or 5 μM CdSO_4_ (gray bars) for 8 days from day 14 after sowing. Values represent the average ± S.E. of 8 biological replicates. Different letters indicate significant differences between conditions (2-way ANOVA; *P* < 0.05).

The extent of endoreplication in leaves 1 and 3 was compared between WT, *smr4/5/7* and *sog1-7* mutant plants grown under control conditions or exposed to 5 μM Cd for 8 days. Representative histograms displaying the ploidy distribution within each experimental condition are shown in Supplementary Figure S2). The obtained results show that the endoreplication index – representing the average number of endocycles per cell – of these leaves was significantly lower in Cd-exposed WT and *smr4/5/7* mutant plants as compared to their control counterparts. This effect was not observed in the *sog1-7* mutant (Figure 2). These data support the lower Cd sensitivity of the *sog1-7* mutant.

### Leaf Cadmium Concentrations Are Similar Between WT, *smr4/5/7* and *sog1-7* Plants

To investigate whether the observed difference in Cd sensitivity was related to the extent of Cd uptake in all genotypes, leaf Cd concentrations were compared between WT, *smr4/5/7* and *sog1-7* mutant plants exposed to 5 μM Cd for 24 h, 72 h or 8 days. Results demonstrate that Cd concentrations in leaf rosettes did not differ between mutant and WT plants at any of the time points investigated (Table 2; Supplementary Table S5).

**TABLE 2:** Cadmium concentrations (mg kg^–1^ DW) in leaf rosettes of hydroponically grown wild-type (WT), *smr4/5/7* and *sog1-7* mutant *A. thaliana* plants exposed to 5 μM CdSO_4_ for 24 h or 72 h from day 19 after sowing or for 8 days from day 14 after sowing.

	WT	*smr4/5/7*	*sog1-7*
24 h	748.19 ± 29.82^ab^	831.03 ± 33.15^a^	616.07 ± 47.89^b^
72 h	1124.67 ± 106.78^a^	1414.87 ± 48.57^a^	1161.43 ± 69.87^a^
8 d	1479.58 ± 479.91^a^	1209.00 ± 259.97^a^	1320.96 ± 49.37^a^

*Values represent the average ± S.E. of 3 biological replicates. Different letters indicate significant differences between conditions (1-way ANOVA per exposure duration; P < 0.05). Cadmium concentrations in non-exposed plants were below the detection limit.*

### Cadmium-Induced Effects on the Expression of Genes Involved in the DNA Damage Response Are Less Pronounced in Leaves of the *sog1-7* Mutant

As negative effects of stress factors on cell cycle progression are an intrinsic part of the DDR, this response was further investigated at the transcriptional level in leaves of the *sog1-7* mutant to gain more insight into the mechanisms underlying its lowered Cd sensitivity. In addition to regulating cell cycle progression, the DDR also coordinates DNA repair and cell death. Therefore, expression levels of selected genes related to each of these three mechanisms (cell cycle regulation, DNA repair and cell death) were compared between leaves of WT and *sog1-7* mutant plants grown under control conditions or exposed to 5 μM Cd for 24 and 72 h (short-term). Expression levels of many of the genes analyzed were considerably increased by Cd in leaves of WT plants after both exposure durations. For most genes, this response was significantly less pronounced in the *sog1-7* mutant after 24 h of exposure and even completely absent after 72 h of exposure (Table 3). Whereas cell death-related genes responded most strongly after 24 h of Cd exposure, the upregulation of genes involved in cell cycle regulation and DNA repair was mainly pronounced after 72 h of exposure (Table 3).

**TABLE 3:**
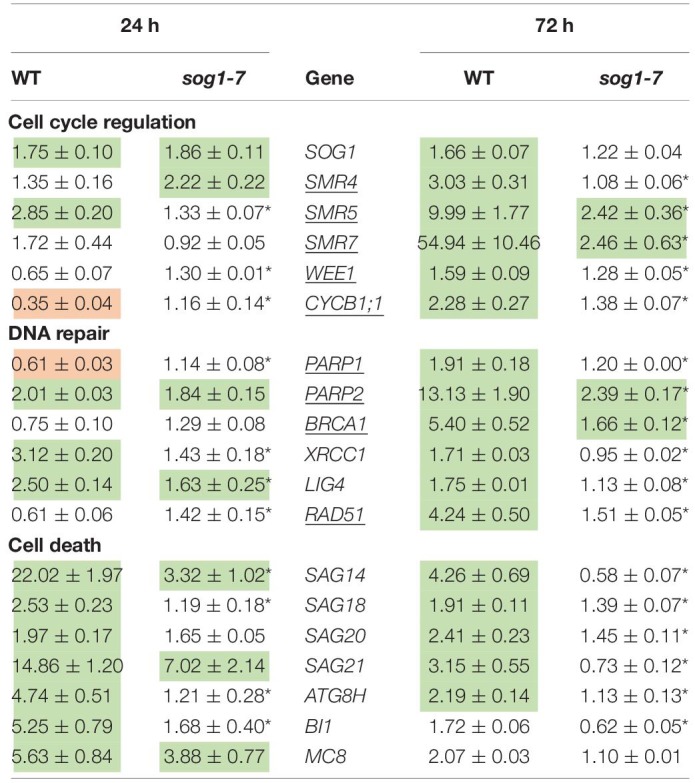
Normalized expression levels of genes involved in the DNA damage response in leaf rosettes of hydroponically grown wild-type (WT) and *sog1-7* mutant *A. thaliana* plants exposed to 5 μM CdSO_4_ for 24 h or 72 h from day 19 after sowing.

Values represent the average ± S.E. of 5 biological replicates and are expressed relative to the average of the same genotype under control conditions at the same time point (set at 1.00). Significant Cd-induced upregulations and downregulations for both normalized and non-normalized data are highlighted in green and red, respectively. Asterisks (*) indicate a significantly different Cd-induced response between both genotypes for both normalized and non-normalized data (2-way ANOVA per exposure duration; P < 0.05). Direct SOG1 target genes according to Ogita et al. (2018) are underlined. ATG8H, autophagy 8H; BI1, Bax inhibitor 1; BRCA1, breast cancer susceptibility 1; CYC, cyclin; MC8, metacaspase 8; PARP, poly(ADP-ribose) polymerase; SOG1, suppressor of gamma response 1; RAD51, DNA repair protein RAD51 homolog 1; SAG, senescence-associated gene; SMR, SIAMESE-related; WEE1, WEE1 kinase homolog; XRCC1, homolog of X-ray repair cross complementing 1.

To determine (1) whether the difference in response between leaves of WT plants and *sog1-7* mutants could also be observed after long-term exposure and (2) whether the absence of a differential response between WT plants and *smr4/5/7* mutants at the cell cycle level coincided with a lack of differential gene expression, a subset of these genes was measured in leaves of *sog1-7* mutant plants after 8 days of exposure and *smr4/5/7* mutants upon 24 and 72 h of exposure. As shown in Table 4, transcript levels of the majority of the genes studied were also significantly upregulated in leaves of the WT after long-term Cd exposure. Again, this response was completely absent in leaves of the *sog1-7* mutant (Table 4). Furthermore, highly similar responses were observed in leaves of the *smr4/5/7* mutant upon short-term exposure (Table 5), which agrees with the fact that Cd sensitivity is unaltered in this genotype.

**TABLE 4:**
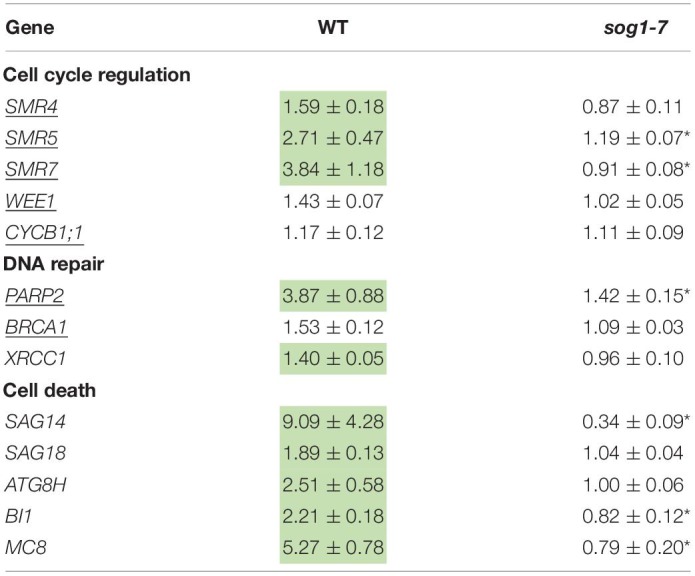
Normalized expression levels of genes involved in the DNA damage response in leaf rosettes of hydroponically grown wild-type (WT) and *sog1-7* mutant *A. thaliana* plants exposed to 5 μM CdSO_4_ for 8 d from day 14 after sowing.

Values represent the average ± S.E. of 5 biological replicates and are expressed relative to the average of the same genotype under control conditions (set at 1.00). Significant Cd-induced upregulations for both normalized and non-normalized data are highlighted in green. Asterisks (*) indicate a significantly different Cd-induced response between both genotypes for both normalized and non-normalized data (2-way ANOVA; P < 0.05). Direct SOG1 target genes according to Ogita et al. (2018) are underlined. ATG8H, autophagy 8H; BI1, Bax inhibitor 1; BRCA1, breast cancer susceptibility 1; CYC, cyclin; MC8, metacaspase 8; PARP, poly(ADP-ribose) polymerase; SAG, senescence-associated gene; SMR, SIAMESE-related; WEE1, WEE1 kinase homolog; XRCC1, homolog of X-ray repair cross complementing 1.

**TABLE 5:**
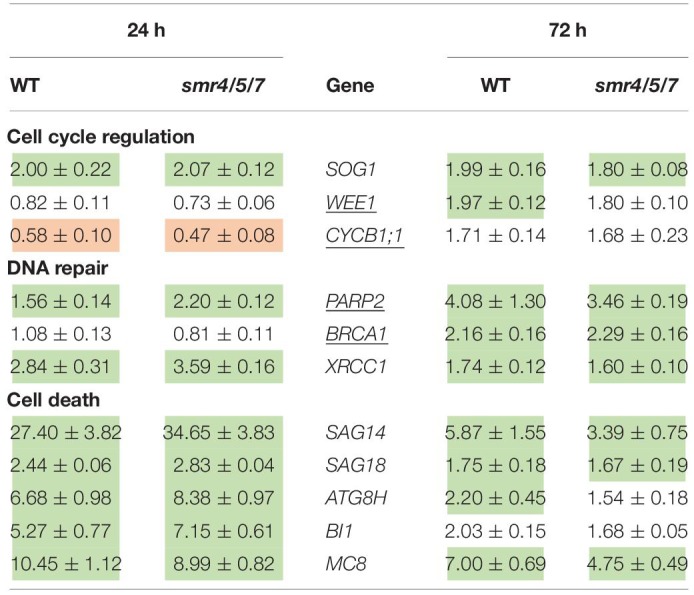
Normalized expression levels of genes involved in the DNA damage response in leaf rosettes of hydroponically grown wild-type (WT) and *smr4/5/7* mutant *A. thaliana* plants exposed to 5 μM CdSO_4_ for 24 h or 72 h from day 19 after sowing.

*Values represent the average ± S.E. of 5 biological replicates and are expressed relative to the average of the same genotype under control conditions at the same time point (set at 1.00). Significant Cd-induced upregulations and downregulations for both normalized and non-normalized data are highlighted in green and red, respectively (2-way ANOVA per exposure duration; P < 0.05). Direct SOG1 target genes according to Ogita et al. (2018) are underlined. ATG8H, autophagy 8H; BI1, Bax inhibitor 1; BRCA1, breast cancer susceptibility 1; CYC, cyclin; MC8, metacaspase 8; PARP, poly(ADP-ribose) polymerase; SOG1, suppressor of gamma response 1; SAG, senescence-associated gene; WEE1, WEE1 kinase homolog; XRCC1, homolog of X-ray repair cross complementing 1.*

### Cadmium-Induced Effects on Expression Levels of Oxidative Stress-Related Genes Are Less Pronounced in Leaves of the *sog1-7* Mutant

Taken together, the data obtained in the first part of this study indicate that the *sog1-7* mutant is less sensitive to acute Cd exposure. As oxidative stress is an important process well known to be involved in Cd-induced stress responses (Cuypers et al., 2011; Gallego et al., 2012), the involvement of SOG1 in oxidative stress and signaling pathways was investigated in the second part of this study. To this end, transcript levels of oxidative stress-related genes were determined in leaves of WT and *sog1-7* mutant plants grown under control conditions or exposed to 5 μM Cd for 24 and 72 h. As shown in Table 6, Cd exposure significantly increased the expression of five oxidative stress hallmark genes in WT leaves. These increases were significantly less pronounced or even completely absent in leaves of the *sog1-7* mutant. Similar responses were observed for *RBOHC* and *RBOHF*, encoding NADPH oxidases involved in superoxide (O_2_^⋅–^) production. Furthermore, Cd-induced effects on transcript levels of several antioxidative genes were significantly less pronounced in leaves of the mutant (Table 6).

**TABLE 6:**
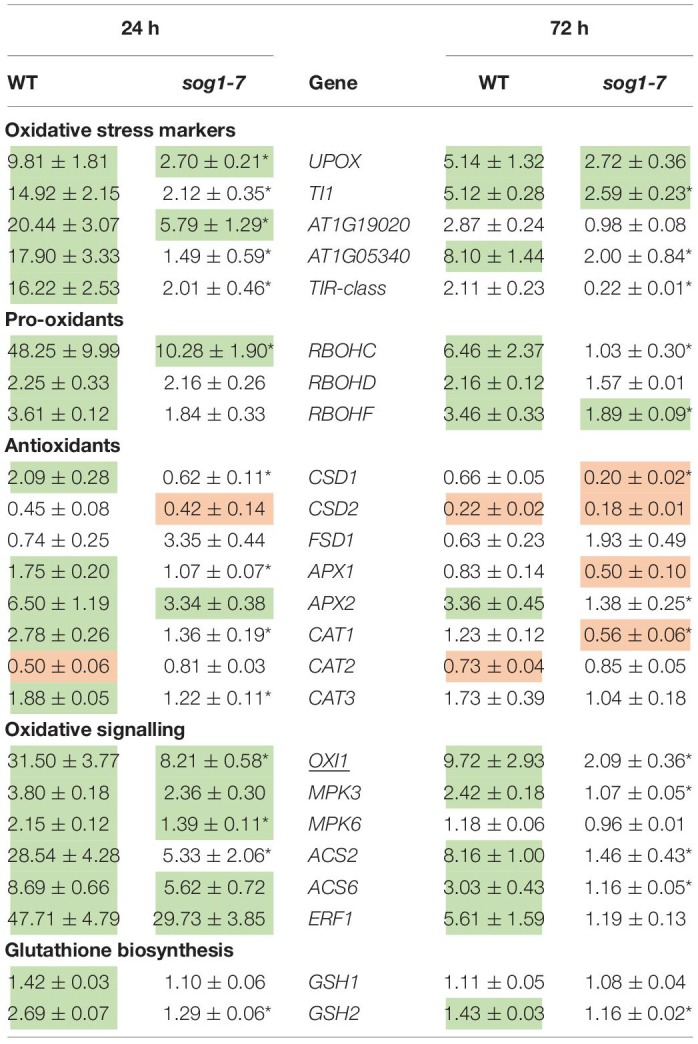
Normalized expression levels of genes involved in the oxidative stress response in leaf rosettes of hydroponically grown wild-type (WT) and *sog1-7* mutant *A. thaliana* plants exposed to 5 μM CdSO_4_ for 24 h or 72 h from day 19 after sowing.

Values represent the average ± S.E. of 5 biological replicates and are expressed relative to the average of the same genotype under control conditions at the same time point (set at 1.00). Significant Cd-induced upregulations and downregulations for both normalized and non-normalized data are highlighted in green and red, respectively. Asterisks (*) indicate a significantly different Cd-induced response between both genotypes for both normalized and non-normalized data (2-way ANOVA per exposure duration; P < 0.05). Direct SOG1 target genes according to Ogita et al. (2018) are underlined. ACS, 1-amino-cyclopropane-1-carboxylate synthase; APX, ascorbate peroxidase; CAT, catalase; CSD, Cu/Zn superoxide dismutase; ERF1, ethylene response factor 1; FSD, Fe superoxide dismutase; GSH1, glutamate-cysteine ligase; GSH2, glutathione synthetase; MPK, mitogen-activated protein kinase; OXI1, oxidative signal-inducible 1; RBOH, respiratory burst oxidase homolog; TI1, trypsin inhibitor 1; TIR1, toll/interleukin receptor 1; UPOX, upregulated by oxidative stress.

As *OXI1* was recently described as a SOG1 target gene (Ogita et al., 2018) and is a key player in the Cd-induced oxidative signaling response (Opdenakker et al., 2012; Schellingen et al., 2015b), the expression of genes involved in the OXI1-MPK signaling pathway was also compared between leaves of both genotypes upon short-term Cd exposure. All genes analyzed were significantly upregulated upon Cd exposure in WT plants, whereas they were significantly less affected in the *sog1-7* mutant (Table 6), pointing toward the involvement of SOG1 in oxidative signaling processes. The expression of the ethylene biosynthesis genes *ACS2* and *ACS6* and the ethylene marker gene *ERF1* showed the same pattern (Table 6). Similarly, transcript levels of GSH biosynthesis genes *GSH1* and *GSH2* were increased by Cd exposure in leaves of WT plants but not in *sog1-7* mutants (Table 6).

The expression of most genes involved in the DNA damage response and the oxidative stress response did not differ between leaves of WT plants and *sog1-7* mutants grown under control conditions (Supplementary Table S6), suggesting that SOG1 is mostly involved in transcriptional regulation in stress-exposed plants.

### Cadmium-Induced Glutathione Biosynthesis Is Delayed in Leaves of the *sog1-7* Mutant

To investigate whether the lack of induction of both GSH biosynthesis genes upon Cd exposure in the *sog1-7* mutant was reflected at the metabolite level, GSH concentrations and redox state were determined in leaves of WT plants and *sog1-7* mutants exposed to Cd for 24 h, 72 h and 8 days.

**FIGURE 3:**
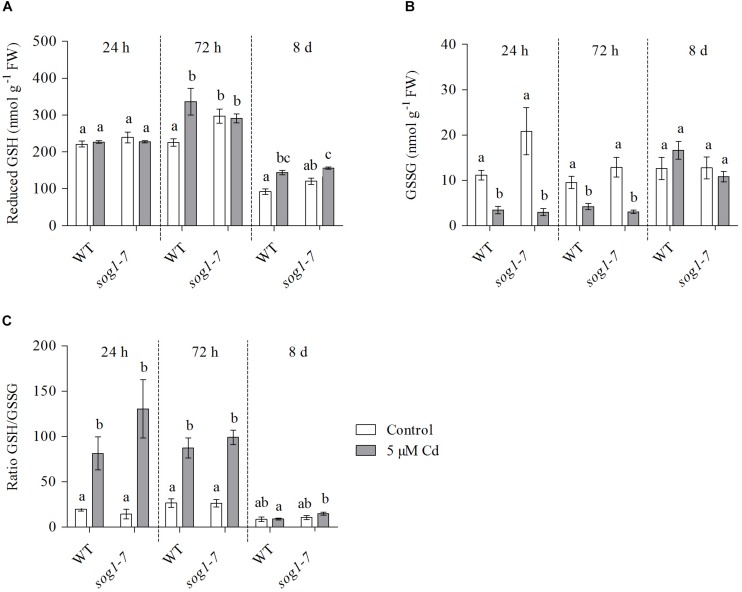
**(A)** Reduced glutathione (GSH) (nmol g^–1^ FW) concentrations, **(B)** glutathione disulfide (GSSG) (nmol g^–1^ FW) concentrations and **(C)** reduced-to-oxidized GSH ratio in leaf rosettes of hydroponically grown wild-type (WT) and *sog1-7* mutant *A. thaliana* plants exposed to 0 (white bars) or 5 μM CdSO_4_ (gray bars) for 24 h, 72 h or 8 days from day 19 after sowing. Values represent the average ± S.E. of at least 4 biological replicates. Different letters indicate significant differences between conditions (2-way ANOVA per exposure duration; *P* < 0.05).

After 24 h of exposure, reduced GSH levels did not change in either genotype (Figure 3A). In contrast, GSSG concentrations significantly decreased (Figure 3B), resulting in an elevated GSH/GSSG ratio (Figure 3C). This response was highly similar between both genotypes. After 72 h of exposure, reduced GSH levels were significantly increased in leaves of WT plants, whereas this effect was not observed in the *sog1-7* mutant (Figure 3A). The negative effect of Cd exposure on GSSG levels persisted after 72 h in both genotypes (Figure 3B). After 8 days of exposure, reduced GSH levels were also significantly increased in leaves of the *sog1-7* mutant, although to a smaller extent than in WT leaves (Figure 3A). In contrast, GSSG concentrations were no longer affected by Cd exposure after this prolonged exposure duration (Figure 3B). It is interesting to notice that reduced GSH concentrations were generally slightly higher in mutant as compared to WT leaves under control conditions (Figure 3A).

### Cadmium-Induced Hydrogen Peroxide Production Is Delayed in Leaves of the *sog1-7* Mutant

**FIGURE 4:**
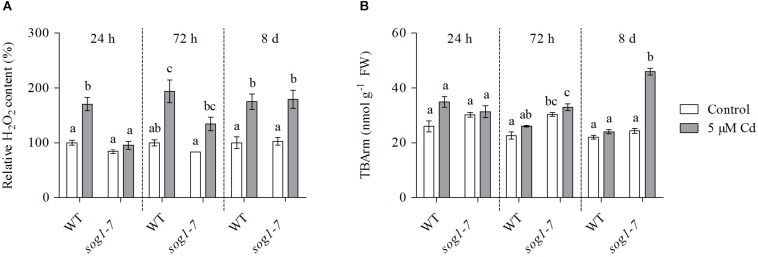
**(A)** Hydrogen peroxide (H_2_O_2_) content (%) relative to wild-type (WT) control and **(B)** TBA-reactive metabolites (TBARm) concentrations (nmol g^–1^ FW) in leaf rosettes of hydroponically grown WT and *sog1-7* mutant *A. thaliana* plants exposed to 0 (white bars) or 5 μM CdSO_4_ (gray bars) for 24 h, 72 h and 8 days from day 19 after sowing. Values represent the average ± S.E. of at least 3 biological replicates. Different letters indicate significant differences between conditions (2-way ANOVA per exposure duration; *P* < 0.05).

To obtain insight into the evolution of ROS levels in both genotypes over time, relative H_2_O_2_ concentrations were determined in leaves of WT and *sog1-7* mutant plants exposed to 5 μM Cd for 24 h, 72 h and 8 days. As shown in Figure 4A, H_2_O_2_ levels were significantly increased after 24 h of Cd exposure in leaves of WT plants, whereas they were unaffected in the *sog1-7* mutant. After 72 h of exposure, H_2_O_2_ concentrations were significantly higher in leaves of Cd-exposed as compared to control plants of both genotypes, although this was less obvious in the mutant (Figure 4A). After a prolonged exposure duration of 8 days, H_2_O_2_ levels were increased to the same extent in both genotypes (Figure 4A). Taken together, these data suggest that the Cd-induced increase of ROS levels is delayed in the *sog1-7* mutant.

### The Extent of Lipid Peroxidation Is Increased in Leaves of the *sog1-7* Mutant After Long-Term Cadmium Exposure

In a final part of this study, the extent of lipid peroxidation was studied in both genotypes, as a measure of the extent of ROS-induced damage to cellular components. To this end, TBArm concentrations – a marker for lipid peroxidation – were measured in leaves of WT and *sog1-7* mutant plants exposed to 5 μM Cd for 24 h, 72 h and 8 days. Whereas the extent of lipid peroxidation was not affected by Cd exposure after 24 and 72 h of exposure (Figure 4B), it was significantly higher in leaves of the *sog1-7* mutant after long-term exposure for 8 days. However, this response was not observed in WT plants (Figure 4B).

## Discussion

**FIGURE 5:**
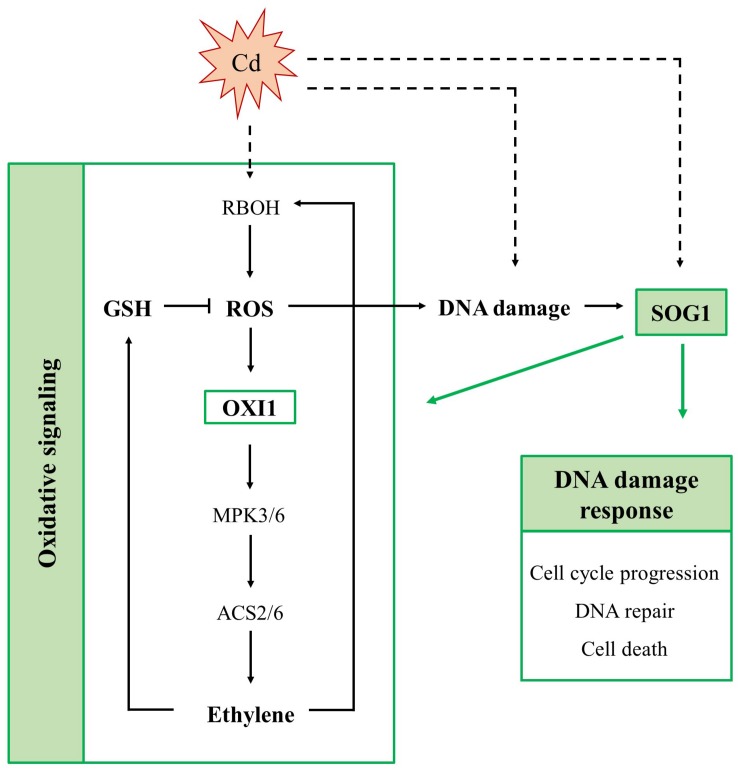
Schematic overview of the involvement of SUPPRESSOR OF GAMMA RESPONSE 1 (SOG1) in acute responses of *A. thaliana* leaves to cadmium (Cd) exposure. SOG1 regulates the Cd-induced DNA damage response, controlling cell cycle progression, DNA repair and cell death. Additionally, it regulates the Cd-induced oxidative signaling pathway, as it affects the extent of reactive oxygen species (ROS) production, ethylene signaling and glutathione (GSH) biosynthesis. The involvement of SOG1 in Cd-induced oxidative signaling possibly results from its direct interaction with the promoter of OXIDATIVE SIGNAL-INDUCIBLE 1 (OXI1). The latter is responsible for activation of a mitogen-activated kinase (MPK) signaling cascade, ultimately inducing ethylene biosynthesis. Whether Cd exposure activates SOG1 through (ROS-induced) DNA damage or through an alternative mechanism remains to be elucidated (indicated in dashed arrows). ACS, 1-amino-cyclopropane-1-carboxylate synthase; RBOH, respiratory burst oxidase homolog.

Cadmium exposure inhibits cell division and endoreplication and increases the expression of SOG1-regulated genes, including *SMR4*, *SMR5* and *SMR7* in leaves of *A. thaliana* (Hendrix et al., 2018). Therefore, the main aim of this study was to further explore the involvement of these CDK inhibitors and their upstream regulator SOG1 in Cd-induced stress responses in *A. thaliana*. To this end, Cd-induced effects were compared between leaves of WT, *smr4/5/7* and *sog1-7* plants exposed to 5 μM Cd in a short-term (24 and 72 h) or long-term (8 days) exposure set-up (Supplementary Figure S1). A schematic overview of the proposed role of SOG1 in the Cd-induced DDR and oxidative signaling is provided in Figure 5.

### The Cadmium-Induced DNA Damage Response in *A. thaliana* Leaves Is Modulated by SOG1

The *sog1-7* mutant was substantially less sensitive to acute Cd exposure in comparison to WT plants, as indicated by the lack of a significant Cd-induced reduction in rosette FW (Figure 1 and Supplementary Table S5), rosette diameter and leaf surface area (Table 1). This mutant was also reported to exhibit a lower aluminum (Al) sensitivity, as shown by a smaller Al-induced reduction of root length (Sjogren et al., 2015). It should be noticed that the *sog1-7* mutant is characterized by significantly smaller leaf rosettes as compared to WT plants under our control conditions (Table 1). The underlying reason for this altered phenotype is currently unclear and requires further investigation.

In contrast to the *sog1-7* mutant, Cd sensitivity of the *smr4/5/7* mutant was highly similar to that of the WT (Figure 1 and Table 1). At the cellular level, Cd exposure inhibited endoreplication in WT leaves (Figure 2), corresponding with results of our previous study (Hendrix et al., 2018). In general, this response was absent in leaves of the *sog1-7* mutant, whereas *smr4/5/7* knockout plants behaved similar to WT plants (Figure 2). The lack of Cd-induced effects on endoreduplication in leaves of *sog1-7* mutants supports a role for SOG1 in cell cycle regulation. Furthermore, it likely contributes to the reduced Cd sensitivity of this genotype. The involvement of SOG1 in the regulation of endoreplication is supported by the fact that cell expansion – often coinciding with endoreplication – induced by the DSB-inducing agent zeocin is suppressed in sepal epidermal cells of the *sog1-1* mutant (Adachi et al., 2011). Furthermore, Chen and Umeda (2015) reported that the zeocin-induced reduction of root length observed in WT plants was absent in *sog1-1* mutants. Root meristem size was decreased in WT roots exposed to zeocin, whereas this effect was not observed in *sog1-1* mutant roots, indicating that SOG1 is required for the zeocin-induced early onset of endoreplication (Chen and Umeda, 2015). A similar result was described by Johnson et al. (2018), showing that the inhibition of DNA replication and cell division observed in the mitotic zone of WT roots shortly after exposure to ionizing radiation was absent in *sog1-1* mutants. Our data indicate that the Cd-induced inhibition of endoreplication in WT plants is also absent in the *sog1-7* mutants (Figure 2), suggesting that under these stress conditions, SOG1 is responsible for the inhibition rather than activation of endoreplication. The fact that Cd-induced effects on endoreplication in the *smr4/5/7* mutant were highly similar to those observed in the WT (Figure 2) suggests that these CDK inhibitors are not responsible for the inhibition of endoreplication observed in leaves of Cd-exposed plants. It cannot be excluded, however, that a (SOG1-regulated) bypass mechanism is activated in the *smr4/5/7* mutant compensating for the lack of functional SMR4, SMR5 and SMR7. This mechanism might involve other proteins of the SMR or KRP families, also involved in regulating cell cycle progression. Furthermore, the highly similar extent of endoreplication between *smr4/5/7* and WT leaves upon Cd exposure might be explained by redundancy between these SMR proteins and WEE1, as the latter is a known SOG1 target (Ogita et al., 2018). Nevertheless, the inhibition of endoreplication in Cd-exposed plants might occur independently of SMR4, SMR5 and SMR7 through SOG1-mediated regulation of other cell cycle-related genes such as *CYCB1;1*. This mitotic cyclin was previously shown to be a direct SOG1 target gene (Ogita et al., 2018). After 24 h of Cd exposure, its expression significantly decreased, whereas it showed an increasing trend upon 72 h of exposure in leaves of WT plants as well as *smr4/5/7* mutants (Table 5). However, in *sog1-7* mutants, no alterations in *CYCB1;1* transcript levels were observed after Cd exposure (Table 3). Whether or not SOG1-dependent regulation of *CYCB1;1* transcription contributes to the observed inhibition of endoreplication in leaves of Cd-exposed plants requires further investigation.

To gain more insight into the involvement of SOG1 in early responses to Cd exposure, transcript levels of genes involved in the DDR were determined in control and Cd-exposed WT and *sog1-7* mutant plants. As previous results indicated that Cd-induced effects on gene expression levels are similar between different leaves (Hendrix et al., 2018), gene expression analyses were performed in entire leaf rosettes. In contrast to Sjogren et al. (2015) who did not observe any effect on *SOG1* gene expression in roots of Al-exposed *Arabidopsis*, we found a significant *SOG1* upregulation in leaves of Cd-exposed seedlings (Table 3). An induction of *SOG1* by Cd exposure was also observed in roots of *A. thaliana* (Cao et al., 2018). Taken together with the fact that SOG1 is known to be regulated at the post-translational level through phosphorylation (Yoshiyama, 2015), this would imply that under certain stress conditions SOG1 could also be regulated at the transcriptional in addition to the post-translational level. Many genes related to the DDR (*i.e.*, related to cell cycle progression, DNA repair and cell death) were induced upon Cd exposure after 24 h and/or 72 h in leaves of WT plants, suggesting the occurrence of Cd-induced DNA damage. Cadmium was previously reported to cause DNA damage in a large range of plant species, as recently reviewed by Huybrechts et al. (2019). The observed Cd-induced DDR was significantly less pronounced or even completely absent in the *sog1-7* mutant (Table 3). These results were confirmed in plants exposed to Cd for 8 days in a long-term exposure set-up (Table 4). In contrast, Cd-induced effects on the transcription of these genes did not differ between leaves of *smr4/5/7* and WT plants (Table 5). The key role of SOG1 in the DDR was also demonstrated by the lack of induction of cell cycle-related and DNA repair genes in *sog1-1* mutants exposed to gamma irradiation (Yoshiyama et al., 2013, 2017) and mitomycin C (Horvath et al., 2017) and *sog1-7* mutants exposed to Al (Sjogren et al., 2015). Furthermore, Ogita et al. (2018) recently reported that several of the genes shown in Table 3 were either directly or indirectly regulated by SOG1 in *A. thaliana* seedlings treated with 15 μM zeocin and contained the consensus SOG1 binding motif. These SOG1 target genes were confirmed by Bourbousse et al. (2018) in seedlings exposed to gamma irradiation.

Our data demonstrate that additional genes could be under the control of SOG1 in leaves of Cd-exposed seedlings, as suggested by their induction in WT plants but not in *sog1-7* mutants. These include *XRCC1* and *LIG4* (involved in DNA repair), several senescence-associated genes (SAGs) and genes involved in autophagy and cell death regulation. Whether these genes are direct SOG1 targets or are indirectly regulated by other transcription factors downstream of SOG1 remains to be investigated. Previous studies have shown that functional SOG1 is required for the induction of cell death in the stem cell region of *A. thaliana* roots exposed to UV-B, gamma irradiation and zeocin (Furukawa et al., 2010; Yoshiyama et al., 2013, 2017). This SOG1-mediated cell death causes the removal of a subset of stem cells and induces a regeneration response in the surrounding root apical meristem. As a consequence, a functional stem cell niche is regained, enabling continued root growth (Johnson et al., 2018). A similar mechanism might be induced by SOG1 upon Cd exposure, as Cd was also shown to induce cell death in the root meristem of *Pisum sativum* (Lehotai et al., 2011) and *A. cepa* (Behboodi and Samadi, 2004).

Taken together, our data indicate that SOG1 is a key player in the Cd-induced DDR in *Arabidopsis* leaves, regulating the expression of a large array of genes involved in cell cycle regulation, DNA repair and cell death-related processes (Figure 5). As transcript levels of some of these genes were slightly increased upon Cd exposure in leaves of *sog1-7* mutant plants – although to a significantly lower extent than in WT plants – it is likely that they are regulated by additional, SOG1-independent pathways. Despite the smaller phenotype of *sog1-7* mutants, the expression of most genes analyzed did not differ between leaves of the WT and the *sog1-7* mutant under control conditions (Supplementary Table S6), indicating that SOG1 is mainly involved in transcriptional regulation under stress conditions. It should be noted, however, that the expression of *SMR4*, *UPOX* and *APX2* was significantly higher in leaves of *sog1-7* mutants as compared to WT plants under control conditions. Although *SMR4* is a known direct SOG1 target gene (Bourbousse et al., 2018; Ogita et al., 2018), these data suggest that additional, SOG1-independent mechanisms are responsible for *SMR4* transcriptional regulation. Furthermore, the elevated transcript levels of the oxidative stress hallmark gene *UPOX* and the stress-responsive gene *APX2* might indicate an increased basal stress level in the *sog1-7* mutant.

### The Cadmium-Induced Oxidative Stress Response in *A. thaliana* Leaves Is Regulated by SOG1

As oxidative stress is an important process involved in plant responses to Cd exposure (Benavides et al., 2005; Cuypers et al., 2010; Gallego et al., 2012), Cd-induced effects on several oxidative stress-related parameters were compared between leaves of WT and *sog1-7* mutant plants to gain additional insight into the role of SOG1 in early Cd-induced stress responses. Cadmium exposure significantly increased the expression of five oxidative stress hallmark genes (Gadjev et al., 2006) in leaves of WT plants exposed for 24 h and/or 72 h (Table 6). These transcriptional inductions were either significantly less pronounced or even completely absent in the *sog1-7* mutant, suggesting a disturbed oxidative stress response in this genotype. Interestingly, *UPOX*, encoding a mitochondrial oxidative stress hallmark gene, was recently reported to be regulated by SOG1 in response to zeocin treatment (Ogita et al., 2018). It is plausible that the oxidative stress hallmark genes are indirectly regulated by SOG1, as the list of SOG1 target genes contains a large number of transcription factors (Bourbousse et al., 2018; Ogita et al., 2018) that could be responsible for their upregulation.

Furthermore, the extent of Cd-induced *RBOHC* and *RBOHF* upregulation was significantly lower in *sog1-7* leaves as compared to WT leaves (Table 6). These genes encode NADPH oxidases located in the plasma membrane and are involved in O_2_^⋅–^ production in Cd-exposed *A. thaliana* leaves (Remans et al., 2010). In general, the expression of antioxidative genes involved in O_2_^⋅–^ and H_2_O_2_ detoxification was also less increased or even decreased in *sog1-7* mutants (Table 6). These data suggest a lower degree of Cd-induced ROS production and antioxidative defense in *sog1-7* mutant leaves, possibly leading to a disturbance in oxidative signaling processes.

A key player in the Cd-induced oxidative signaling pathway is OXI1. As proposed by Schellingen et al. (2015b), Cd exposure increases ROS production through the activation of NADPH oxidases. Increased ROS levels are then perceived by OXI1, which subsequently activates MPK3 and MPK6 through phosphorylation. Interestingly, OXI1 was recently shown to contain a SOG1 binding motif (Ogita et al., 2018) and could hence function as a link between SOG1 and Cd-induced oxidative signaling (Figure 5). Our results showed that Cd exposure significantly increased transcript levels of *OXI1*, *MPK3* and *MPK6* after 24 and 72 h of exposure in WT plants. This effect was significantly less pronounced or even completely abolished in the *sog1-7* mutant (Table 6). As MPK3 and MPK6 are known to affect the transcription and activity of ACS2 and ACS6, involved in ethylene biosynthesis, transcript levels of both genes were also determined and *ERF1* gene expression was measured as a marker for ethylene signaling. Results showed a pattern highly similar to that of *OXI1*, *MPK3* and *MPK6*, with significantly less pronounced Cd-induced upregulations in the *sog1-7* mutant (Table 6). In addition, the expression of GSH biosynthesis genes *GSH1* and *GSH2* was determined, as GSH is a key player in plant responses to Cd stress through its antioxidative and Cd-chelating functions (Jozefczak et al., 2012). As shown in Table 6, *GSH1* and *GSH2* transcript levels were significantly upregulated upon Cd exposure in WT leaves but not in *sog1-7* mutant leaves.

To determine whether differences in Cd-induced effects on the expression of GSH biosynthesis genes were also reflected at the metabolite level, reduced and oxidized GSH concentrations were determined in leaves of WT and *sog1-7* mutant *A. thaliana* plants exposed to Cd for 24 h, 72 h and 8 days. Concentrations of GSSG showed an immediate decrease upon Cd exposure in leaves of both genotypes (Figure 3B). This response is often observed in leaves of Cd-exposed *A. thaliana* and generally coincides with elevated transcript levels of glutathione reductase (GR), suggesting an increased reduction of GSSG to GSH (Jozefczak et al., 2014; Keunen et al., 2015; Schellingen et al., 2015a). In addition, Cd-induced increases in reduced GSH levels were observed from 72 h of exposure in WT plants, but were delayed in *sog1-7* mutants (Figure 3A). These data agree with the lack of a Cd-induced upregulation of *GSH1* and *GSH2* in the mutant after short-term exposure (Table 6). A lack of transcriptional induction of the GSH biosynthesis genes and the absence of increases in GSH concentrations after exposure to 5 μM Cd was also observed in leaves of the ethylene insensitive *etr1-1* and *ein2-1* mutants (Schellingen et al., 2015b). Interestingly, these mutants showed a decreased Cd sensitivity as compared to the WT (Schellingen et al., 2015b). Our data indicate that a similar connection between ethylene and GSH metabolism might exist in the leaves of *sog1-7* mutant plants, as both the expression of ethylene-related genes and reduced GSH concentrations were less affected by Cd exposure in this genotype. Taken together, these data point toward a key role for SOG1 in acute Cd-induced oxidative signaling (Figure 5). The reduced Cd-induced effects on oxidative signaling in leaves of the *sog1-7* mutant could either be explained by a lower degree of ROS production in the mutant or by a reduced ability of the mutant to induce oxidative signaling pathways.

Therefore, Cd-induced effects on leaf H_2_O_2_ concentrations were compared between WT and *sog1-7* plants exposed to 5 μM Cd for 24 h, 72 h and 8 days. Cadmium-induced increases in H_2_O_2_ levels were delayed in the mutant (Figure 4A). Chen and Umeda (2015) reported that root H_2_O_2_ levels were increased by 24 h of zeocin exposure in WT *A. thaliana* plants, but not in a *sog1-1* mutant. This response corresponded with the absence of a zeocin-induced upregulation of *FLAVIN-DEPENDENT MONOOXYGENASE 1* (*FMO1*), involved in ROS production in roots (Chen and Umeda, 2015). The fact that H_2_O_2_ production did not increase in leaves of the mutant after 24 h of Cd exposure (Figure 4A) possibly underlies the absence of a transcriptional induction of the oxidative signaling pathway (Table 6). As a result, an induction of a clear defense response is absent in *sog1-7* mutants after 72 h of Cd exposure. Therefore, increased levels of oxidative damage might be expected in the mutant after prolonged Cd exposure, since increased H_2_O_2_ concentrations were observed at this time point. This was confirmed by a significant increase in TBArm in leaves of mutant but not WT plants after 8 days of exposure (Figure 4B). Taken together, our work shows that Cd-induced ROS production is delayed in leaves of the *sog1-7* mutant. As a consequence, oxidative signaling and a clear defense response are insufficiently induced, resulting in an increased extent of oxidative damage after prolonged exposure.

In conclusion, our data strongly support the involvement of SOG1 in the Cd-induced DDR in leaves of *A. thaliana*. Interestingly, the obtained results also imply an additional role for SOG1 in regulating the oxidative stress response in Cd-exposed plants. The involvement of SOG1 in this process has never been described before and suggests that this protein functions in additional pathways besides the DNA damage response. Therefore, we propose that SOG1 might function as a general integrator of stress responses in plants exposed to Cd and possibly additional stress factors as well. A Cd-induced transcriptional upregulation of *SOG1* was also reported in *A. thaliana* roots (Cao et al., 2018). Hence, future research should aim to identify whether a similar SOG1-mediated response is activated by Cd exposure in roots.

## Data Availability Statement

The datasets generated for this study are available on request to the corresponding author.

## Author Contributions

SH, EK, and AC envisioned the project and designed the experiments. SH, VI, and IP carried out the experiments. TE generated the *smr4/5/7* mutant. SH analyzed the data and together with AC, wrote the manuscript. MH, LD, NH, and JV revised the article critically for important intellectual content. All authors have read and approved the manuscript.

## Conflict of Interest

The authors declare that the research was conducted in the absence of any commercial or financial relationships that could be construed as a potential conflict of interest.
