# Supplementary material for: Suppressor of Gamma Response 1 Modulates the DNA Damage Response and Oxidative Stress Response in Leaves of Cadmium-Exposed Arabidopsis thaliana
Source: Front Plant Sci. 2020 Apr 3;11:366. doi: 10.3389/fpls.2020.00366 (PMC7145961; doi:10.3389/fpls.2020.00366)
Supplement: Supplementary file 1 [file Data_Sheet_1.pdf]

frontiers

Supplementary Material

1 Supplementary Figures

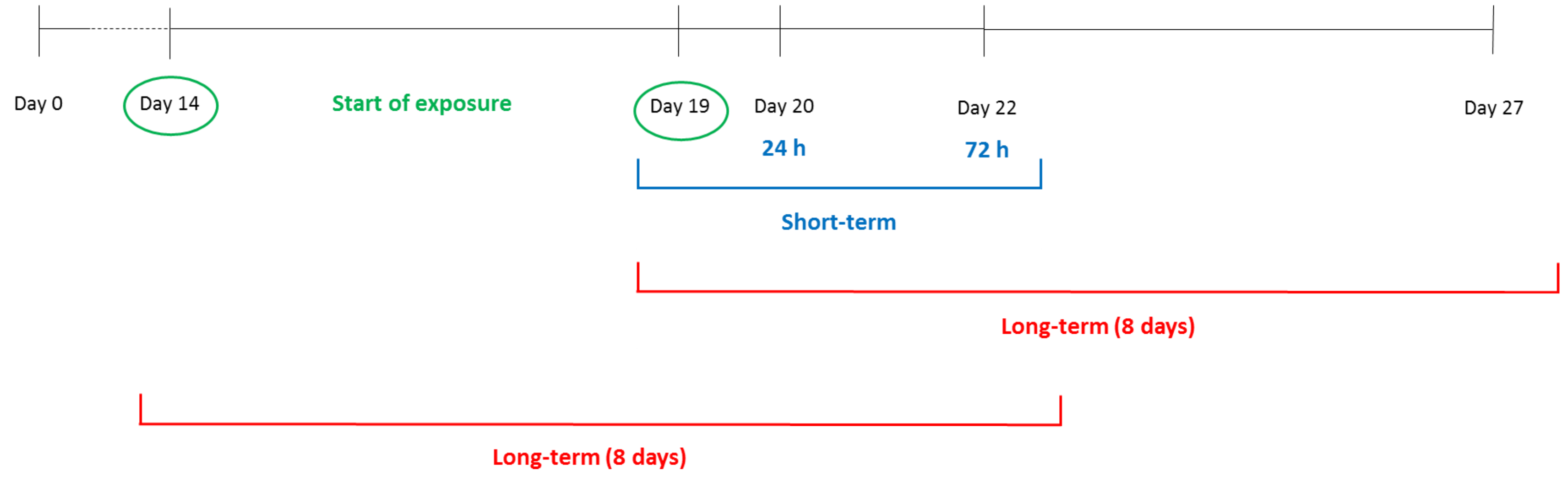

Supplementary Figure S1. Visual representation of the different Cd exposure set-ups employed in this study. Plants were exposed to 5 µM $CdSO_4$ at either 14 or 19 days after sowing and harvested after 24 or 72 h (*i.e.* short-term exposure) or 8 days (*i.e.* long-term exposure).

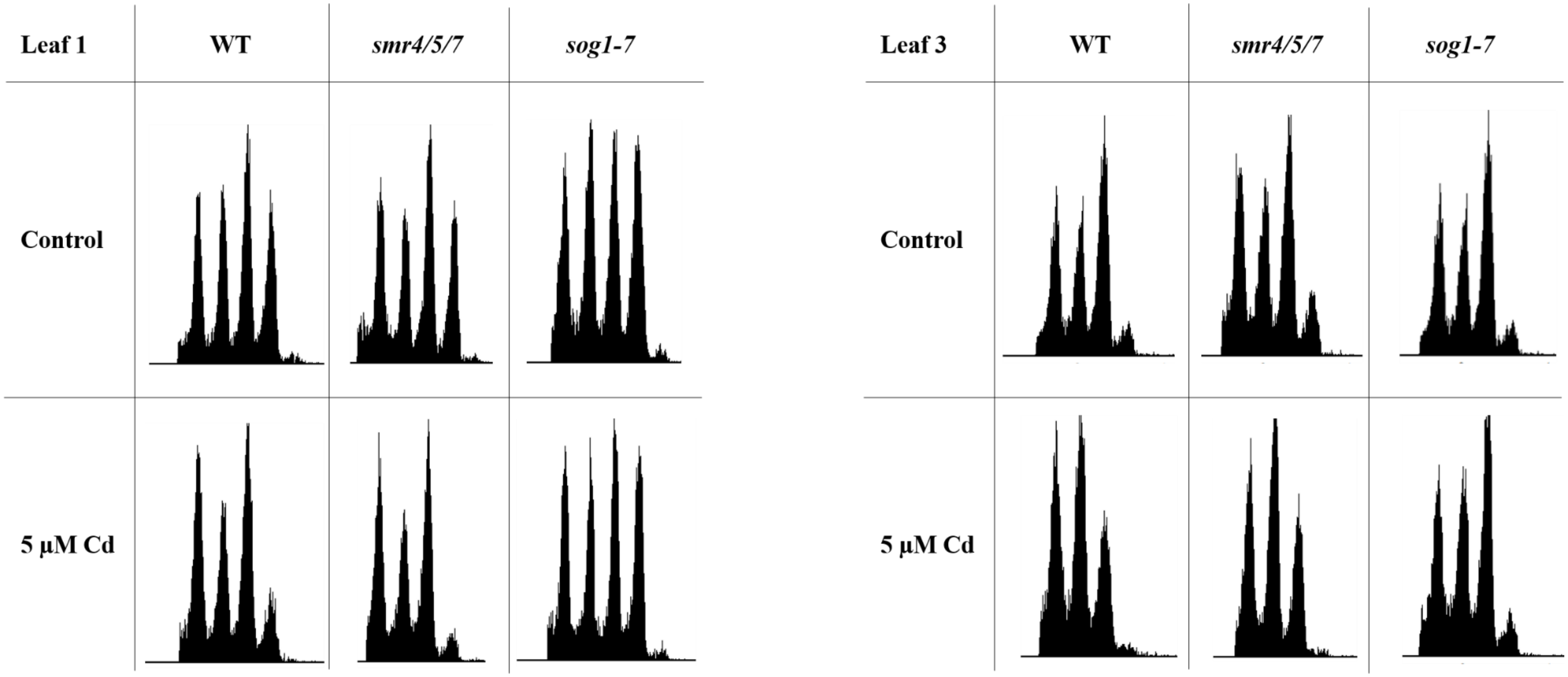

Supplementary Figure S2. Ploidy distribution in leaf 1 and leaf 3 of hydroponically grown wild-type (WT), *smr4/5/7* and *sog1-7* mutant *A. thaliana* plants exposed to 0 or 5 µM $CdSO_4$ for 8 days from day 14 after sowing.

2 Supplementary Tables

Supplementary Table S1. Sequences of forward and reverse primers (5'-3') used to generate a construct targeting two distinct sites in the *SMR4* gene.

Primer	Sequence (5'-3')
SMR4_g1_CRISPR_FW	ATTGAAGCACGATGTATAGGATTC
SMR4_g1_CRISPR_REV	AAACGAATCCTATACATCGTGCTT
SMR4_g2_CRISPR_FW	ATTGAACTTCTTCCTCGGCGGCGG
SMR4_g2_CRISPR_REV	AAACCCGCCGCCGAGGAAGAAGTT

Supplementary Table S2. Genotyping method and sequences of forward and reverse primers (5'-3') used for verification of *smr4/5/7* and *sog1-7* mutant genotypes. DNA was extracted using the Plant Phire Direct PCR Kit (Thermo Fisher Scientific) and PCR was performed using the Phire Plant Direct PCR Kit for T-DNA insertions or Phusion High-Fidelity PCR Kit (Thermo Fisher Scientific) for single nucleotide mutations. The T-DNA primer was used in combination with the reverse primer.

Gene	Mutation type	Genotyping method	Forward primer (5'-3')	Reverse primer (5'-3')	T-DNA primer (5'-3')
SMR4	Nucleotide insertion	DNA sequencing	ATGTAACCGCCTCTTCTCAG	GAACACAAGCCAACAAATTTCG	-
SMR5	T-DNA insertion	PCR	GAACGAACAAAAGTGAGCTCG	TTTCCCAACCTGACAGAAAAC	ATTTTGCCGATTTCGGAAC
SMR7	T-DNA insertion	PCR	AAAATCGATAACTAAAACGAACCG	AGGCCTTCAATATAGCCCATG	
SOG1	Nucleotide substitution	CAPS PCR and DdeI restriction	CAGAGACTTCCTGTTCAG	CAATCAAAGCTTATAGCACAG	-

Supplementary Table S3. Forward and reverse primers used for RT-qPCR. E-E-jn: exon-exon junction; UTR: untranslated region; *: primer concentration of 600 nM instead of 300 nM, **: primer concentration of 900 nm instead of 300 nM.

Gene	Locus	Forward primer (5'-3')	Reverse primer (5'-3')	Exon location	Amplicon size
ACT2	*AT3G18780*	CTTGCACCAAGCAGCATGAA	CCGATCCAGACACTGTACTTCCTT	Exon 2	68 bp
MON1	*AT2G28390*	AACTCTATGCAGCATTTGATCCACT	TGATTGCATATCTTTATCGCCATC	Exon 13 and 14	61 bp
YLS8	*AT5G08290*	TTACTGTTTCGGTTGTTCTCCATTT	CACTGAATCATGTTCGAAGCAAGT	UTR	61 bp
SOG1	*AT1G25580*	AGTGGTGTGGAAGAGCAACC	GCAATCCTGGCCAATCATCAA	Exon 2 and 3	92 bp
SMR4	*AT5G02220*	TGATGGTGGTGAGAAAACGAGA	TCTCTTCGAGGCTGTGCGTAG	Exon 1	91 bp
SMR5	*AT1G07500*	CAGCATATCCGCCTTGTCCA	CTGCTACCACCGAGAAGAACAAGT	Exon 1 and 2	91 bp
SMR7 *	*AT3G27630*	ACATCGATTCGGGCTTCACTAA	CCGTGGGAGTGATACAAATTCC	Exon 1	91 bp
WEE1	*AT1G02970*	TCAGAACTTGATGAGCGGCT	TGTGAAACTCTCCTGGCGAC	Exon 2 and E3-E4-jn	112 bp
CYCB1;1 *	*AT4G37490*	CACGATCTCAAAATCCCACGC	TTCCCAGCCACTTTCTTCGG	Exon 2 and 3	97 bp
PARP1	*AT2G31320*	TGCATTGGGAGAAATACATGAGC	CCGAGCCCTTTGGTCGAG	Exon 17 and 18	84 bp
PARP2	*AT4G02390*	ATCGGAGGTGATTGATCGGTATG	AAATCATGAGGTATCACTGTGTAGAACTCT	Exon 8 and 9	81 bp
BRCA1	*AT4G21070*	GTGAACCTGTCTCTGCGGAT	TCCGGCTTCTTGTCAACTCC	Exon 8 and 9	138 bp
XRCC1	*AT1G80420*	TGGGCCAGGGATGACCTAAG	CCGCAGCTATTCGCTTGATTT	Exon 5 and 6	91 bp
LIG4	*AT5G57160*	TGATGTATCGGATATCAAGGGCA	GAATGGGACCGAGGCACG	Exon 19 and 20	81 bp
RAD51	*AT5G20850*	GTCCAACAACAAGACGATGAAGAA	AACAGAAGCAATACCTGCTGCC	Exon 1 and E1-E2-jn	81 bp
SAG14	*AT5G20230*	GAGACCTATGGACCCCGAGT	ACAACTGCCACATCATGCCT	Exon 1 and 2	111 bp
SAG18	*AT1G71190*	TCCCACAATCCCTCAATCTC	ACCAATAAGGCCGATGATGA	Exon 1 and 2	121 bp
SAG20	*AT3G10985*	AACAGCCACGTCAGGAGAAT	ACCGCGTTTAAAACAGCAAC	UTR	110 bp
SAG21	*AT4G02380*	TCTCGTGAACTCTCCAATGCT	GCAACAGCTCCACTTCTTCC	Exon 1 and 2	95 bp
ATG8H	*AT3G06420*	TGCAGTTAGATCCATCCAAAGCT	ACCCGTCTTCTTCCTTGAAAGT	Exon 4 and 5	108 bp
BI1	*AT5G47120*	GCTTCTGTTGGCCCCTTGAT	CGTCTTGCTAACATTGCTGCT	Exon 3 and 4	122 bp
MC8	*AT1G16420*	TGGACATGGCACGAGAATCC	ACCTTCTTTTACACGTGACACCA	Exon 1 and 2	139 bp
UPOX	*AT2G21640*	GACTTGTTTCAAAAACACCATGGAC	CACTTCCTTAGCCTCAATTTGCTTC	Exon 1 and 2	91 bp
TI1	*AT2G43510*	ATGGCAAAGGCTATCGTTTCC	CGTTACCTTGCGCTTCTATCTCC	Exon 1 and 2	91 bp
Unknown	*AT1G19020*	GAAAATGGGACAAGGGTTAGACAAA	CCCAACGAAAACCAATAGCAGA	Exon 1	92 bp

Supplementary Table S3 (continued).

Gene	Locus	Forward primer (5'-3')	Reverse primer (5'-3')	Exon location	Amplicon size
TIR-class	*AT1G57630*	ACTCAAACAGGCGATCAAAGGA	CACCAATTCGTCAAGACAACACC	Exon 1	91 bp
RBOHC	*AT5G51060*	TCACCAGAGACTGGCACAATAAA	GATGCTCGACCTGAATGCTC	Exon 6 and 7	101 bp
RBOHD	*AT5G47910*	AACTCTCCGCTGATTCCAACG	TGGTCAGCGAAGTCTTTAGATTCCT	Exon 1 and 2	91 bp
RBOHF	*AT1G64060*	GGTGTCATGAACGAAGTTGCA	AATGAGAGCAGAACGAGCATCA	Exon 11 and 12	99 bp
CSD1	*AT1G08830*	TCCATGCAGACCCTGATGAC	CCTGGAGACCAATGATGCC	Exon 5 and E6-E7-jn	102 bp
CSD2	*AT2G28190*	GAGCCTTTGTGGTTCACGAG	CACACCACATGCCAATCTCC	Exon 6 and E7-E8-jn	101 bp
FSD1	*AT4G25100*	CTCCCAATGCTGTGAATCCC	TGGTCTTCGGTTCTGGAAGTC	Exon 4 and E6-E7-jn	101 bp
APX1	*AT1G07890*	TGCCACAAGGATAGGTCTGG	CCTTCCTTCTCTCCGCTCAA	Exon 5 and 6	101 bp
APX2	*AT3G09640*	TTGCTGTTGAGATCACTGGAGGA	TGAGGCAGACGACCTTCAGG	Exon 3 and 4	91 bp
CAT1	*AT1G20630*	AAGTGCTTCATCGGGAAGGA	CTTCAACAAAACGCTTCACGA	E5-E6-jn and exon 7	103 bp
CAT2	*AT4G35090*	AACTCCTCCATGACCGTTGGA	TCCGTTCCCTGTCGAAATTG	Exon 2 and 3	91 bp
CAT3	*AT1G20620*	TCTCCAACAACATCTCTTCCCTCA	GTGAAATTAGCAACCTTCTCGATCA	Exon 2 and 3	91 bp
OXI1	*AT3G25250*	TAGAGGATCGAACCGGAAAG	GACCCTTGATTTCCTCAACG	Exon 2	149 bp
MPK3	*AT3G45640*	GACGTTTGACCCCAACAGAA	TGGCTTTTGACAGATTGGCTC	Exon 5 and 6	103 bp
MPK6	*AT2G43790*	TAAGTTCCCGACAGTGCATCC	GATGGGCCAATGCGTCTAA	Exon 5 and 6	100 bp
ACS2	*AT1G01480*	CATGTTCTGCCTTGCGGATC	ACCTGTCCGCCACCTCAAGT	Exon 3 and 4	91 bp
*ACS6 ** *	*AT4G11280*	TTAGCTAATCCCGGCGATGG	ACAAGATTCACTCCGGTTCTCCA	Exon 3 and 4	92 bp
*ERF1 * *	*AT3G23240*	TCCTCGGCGATTCTCAATTTT	CAACCGGAGAACAACCATCCT	Exon 1	91 bp
GSH1	*AT4G23100*	CCCTGGTGAACTGCCTTCA	CATCAGCACCTCTCATCTCCA	Exon 5 and 6	101 bp
GSH2	*AT5G27380*	GGACTCGTCGTTGGTGACAA	TCTGGGAATGCAGTTGGTAGC	Exon 11 and 12	101 bp

Supplementary Table S4. Reverse transcription quantitative PCR parameters according to the Minimum Information for publication of Quantitative real-time PCR Experiments (MIQE) guidelines derived from Bustin et al. (2009).

**Sample/Template**	
Source	*Arabidopsis thaliana* leaves in a hydroponic culture
Method of preservation	Harvest in liquid nitrogen, storage at -80 °C
Storage time (if appropriate)	Maximum two weeks
Handling	Frozen
Extraction method	Columns: RNAqueous™ Kit
RNA: DNA-free	Turbo DNA-*free* ™ Kit
	Use of intron-spanning primers whenever possible
	Verification of single peak on dissociation curves
Concentration	NanoDrop® ND-1000 spectrophotometer
RNA: integrity	Microfluidics: Agilent 2100 Bioanalyzer with Agilent RNA 6000 Nano Kit
**Assay optimisation/validation**	
Accession number	Table S3
Amplicon details	Exon location and amplicon size: Table S3
Primer sequence	Table S3
In silico	Primer-BLAST (http://www.arabidopsis.org/Blast/index.jsp)
Empirical	Primer concentrations of 300 nM unless stated otherwise (Table S3)
	Annealing temperature of 60 °C
Priming conditions	Combination of oligo-dT primers and random hexamers
PCR efficiency	Dilution curves (slope, deviation)
Linear dynamic range	Samples are within the range of the efficiency curve
**RT and qPCR**	
Protocols	Turbo DNA-*free* ™ Kit
	PrimeScript™ RT Reagent Kit
	Quantinova™ SYBR® Green PCR Kit
	As described in the Materials and methods section
Reagents	As described in the materials and methods section
NTC	Cq and dissociation curve verification
**Data analysis**	
Specialist software	7500 Fast System Sequence Detection Software, version 1.4.0
Statistical justification	As described in the Materials and methods section and Table legends
Transparent, validated normalisation	Minimum three references genes selected using the GrayNorm algorithm
	As described in the Materials and methods section

Supplementary Table S5. Rosette fresh weight (mg) and Cd concentrations (mg kg^{-1} DW) in leaf rosettes of hydroponically grown wild-type (WT) and *sog1-7* mutant *A. thaliana* plants exposed to 0 or 5 µM $CdSO_4$ for 8 days from day 19 after sowing. Values represent the average ± S.E. of at least 4 biological replicates. Different letters indicate significant differences between conditions (fresh weight: 2-way ANOVA; Cd concentration: 1-way ANOVA; $P < 0.05$). N.D.: not detected.

	**WT**		***sog1-7***	
	**Control**	**5 µM Cd**	**Control**	**5 µM Cd**
**FW (mg)**	162.06 ± 11.63[a]	108.56 ± 5.42[b]	107.56 ± 6.13[b]	92.28 ± 3.98[b]
**Cd (mg kg^{-1} DW)**	N.D.	1540.84 ± 179.94	N.D.	1435.28 ± 101.05

Supplementary Table S6. Normalized expression levels of genes involved in the DNA damage response and oxidative stress response in leaf rosettes of hydroponically grown wild-type (WT) and *sog1-7* mutant *A. thaliana* plants grown under control conditions for 20 days. Values represent the average ± S.E. of 5 biological replicates and are expressed relative to the average of the wild-type under control conditions at the same time point (set at 1.00). Significant upregulations compared to the wild-type are highlighted in green (Student's t-test; P < 0.05). *ACS*: 1-amino-cyclopropane-1-carboxylate synthase; *APX*: ascorbate peroxidase; *ATG8H*: autophagy 8H; *BI1*: Bax inhibitor 1; *BRCA1*: breast cancer susceptibility 1; *CAT*: catalase; *CSD*: Cu/Zn superoxide dismutase; *CYC*: cyclin; *ERF1*: ethylene response factor 1; *FSD*: Fe superoxide dismutase; *GSH1*: glutamate-cysteine ligase; *GSH2*: glutathione synthetase; *MC8*: metacaspase 8; *MPK*: mitogen-activated protein kinase; *OXI1*: oxidative signal-inducible 1; *PARP*: poly(ADP-ribose) polymerase; *RBOH*: respiratory burst oxidase homolog; *SOG1*: suppressor of gamma response 1; *RAD51*: DNA repair protein RAD51 homolog 1; *SAG*: senescence-associated gene; *SMR*: SIAMESE-related; *TI1*: trypsin inhibitor 1; *TIR1*: toll/interleukin receptor 1; *UPOX*: upregulated by oxidative stress; *WEE1*: WEE1 kinase homolog; *XRCC1*: homolog of X-ray repair cross complementing 1.

**Gene**	**WT**	***sog1-7***
**Cell cycle regulation**		
SOG1	1.00 ± 0.08	1.13 ± 0.04
SMR4	1.00 ± 0.12	1.89 ± 0.30
SMR5	1.00 ± 0.09	1.28 ± 0.02
SMR7	1.00 ± 0.06	0.87 ± 0.02
WEE1	1.00 ± 0.05	1.12 ± 0.04
CYCB1;1	1.00 ± 0.04	0.92 ± 0.05
**DNA repair**		
PARP1	1.00 ± 0.02	0.96 ± 0.04
PARP2	1.00 ± 0.06	1.00 ± 0.05
BRCA1	1.00 ± 0.01	1.10 ± 0.07
XRCC1	1.00 ± 0.09	1.11 ± 0.09
LIG4	1.00 ± 0.05	1.04 ± 0.04
RAD51	1.00 ± 0.03	1.19 ± 0.13
**Cell death**		
SAG14	1.00 ± 0.19	1.15 ± 0.21
SAG18	1.00 ± 0.08	0.99 ± 0.12
SAG20	1.00 ± 0.09	0.76 ± 0.10
SAG21	1.00 ± 0.21	0.84 ± 0.11
ATG8H	1.00 ± 0.11	1.04 ± 0.18
BI1	1.00 ± 0.19	1.56 ± 0.33
MC8	1.00 ± 0.25	1.10 ± 0.28

Supplementary Table S6 (continued).

Gene	WT	*sog1-7*
**Oxidative stress markers**		
UPOX	1.00 ± 0.07	1.48 ± 0.14
TI1	1.00 ± 0.21	1.09 ± 0.18
AT1G19020	1.00 ± 0.24	0.93 ± 0.17
AT1G05340	1.00 ± 0.25	2.43 ± 0.85
TIR-class	1.00 ± 0.35	2.59 ± 0.95
**Pro-oxidants**		
RBOHC	1.00 ± 0.14	0.73 ± 0.22
RBOHD	1.00 ± 0.10	0.89 ± 0.13
RBOHF	1.00 ± 0.07	1.20 ± 0.24
**Antioxidants**		
CSD1	1.00 ± 0.15	1.02 ± 0.15
CSD2	1.00 ± 0.04	0.85 ± 0.16
FSD1	1.00 ± 0.04	0.74 ± 0.28
APX1	1.00 ± 0.09	1.08 ± 0.09
APX2	1.00 ± 0.10	3.43 ± 0.74
CAT1	1.00 ± 0.06	1.30 ± 0.13
CAT2	1.00 ± 0.09	1.01 ± 0.07
CAT3	1.00 ± 0.03	0.96 ± 0.07
**Oxidative signaling**		
OXI1	1.00 ± 0.03	1.05 ± 0.21
MPK3	1.00 ± 0.17	1.01 ± 0.04
MPK6	1.00 ± 0.04	1.03 ± 0.07
ACS2	1.00 ± 0.28	0.99 ± 0.19
ACS6	1.00 ± 0.10	1.18 ± 0.15
ERF1	1.00 ± 0.25	0.73 ± 0.14
**Glutathione biosynthesis**		
GSH1	1.00 ± 0.04	0.92 ± 0.04
GSH2	1.00 ± 0.06	0.92 ± 0.03
